# Identification of the Group III *WRKY* Subfamily and the Functional Analysis of *GhWRKY53* in *Gossypium hirsutum* L.

**DOI:** 10.3390/plants10061235

**Published:** 2021-06-17

**Authors:** Dongjie Yang, Yuanyuan Liu, Hailiang Cheng, Qiaolian Wang, Limin Lv, Youping Zhang, Guoli Song, Dongyun Zuo

**Affiliations:** 1 Institute of Cotton Research of Chinese Academy of Agricultural Sciences, Anyang 455000, China; 82101185040@caas.cn (D.Y.); yuanyl4956@163.com (Y.L.); pser2010@163.com (H.C.); zuodongyun@caas.cn (Q.W.); llm0372@126.com (L.L.); zyp547550790@163.com (Y.Z.); 2 Zhengzhou Research Base, State Key Laboratory of Cotton Biology, Zhengzhou University, Zhengzhou 450001, China

**Keywords:** cotton, gene family, fiber initiation, putative group III *WRKY* genes, expression pattern

## Abstract

WRKY transcription factors had multiple functions in plant secondary metabolism, leaf senescence, fruit ripening, adaptation to biotic and abiotic stress, and plant growth and development. However, knowledge of the group III *WRKY* subfamily in fiber development in upland cotton (*Gossypium hirsutum* L.) is largely absent. Previous studies have shown that there were 21 putative group III *WRKY* members in *G. hirsutum* L. These putative amino acid sequences from the III WRKY group were phylogenetically clustered into three clades. Multiple alignment, conservative motif analysis, and gene structure analysis showed that the members clustered together in the phylogenetic tree had similar motifs and gene structures. Expression pattern analysis revealed that variation in the expression levels of these genes in different tissues and fiber development stages. To better understand the functions of putative group III *WRKY* genes in *G. hirsutum* L., we selected the cotton fiber initiation-related gene *GhWRKY53* for cloning and functional identification. The subcellular localization experiment of *GhWRKY53* in *Nicotiana tabacum* leaves showed that it was located in the nucleus. The heterologous expression of *GhWRKY53* in *Arabidopsis thaliana* could significantly increase the density of trichomes. Twelve proteins that interacted with GhWRKY53 were screened from the cotton fiber cDNA library by yeast two-hybrid experiment. This study findings lay a foundation for further research on the role of the *GhWRKY53* during cotton fiber development and provide a new insight for further studying putative group III *WRKY* genes in *G. hirsutum* L. Our research results also provide vital information for the genetic mechanism of high-quality cotton fiber formation and essential genetic resources for cotton fiber quality improvement.

## 1. Introduction

The name of the *WRKY* gene family is derived from the conserved protein structural feature of WRKY proteins, the WRKY domain [1,2]. The WRKY domain composed of 60 amino acid residues, and its amino-terminal isa highly conserved protein domain consisting of seven amino acid residues (WRKYGQK). The carboxyl-terminal is C_2_H_2_ (C-X_4-5_-C-X_22-23_-H-X_1_-H) or C_2_HC (C-X_7_-C-X_23_-H-X_1_-C) zinc finger motif. Some WRKY proteins contain a glutamate-rich region, a proline-rich region, and a leucine zipper structure [3]. According to the WRKY domains and zinc finger motifs, the WRKY transcription factor family can be divided into three subgroups: I, II and III. Subgroup I contain two WRKY domains, each domain have a C_2_H_2_ zinc finger motif. In comparison, subgroup II and subgroup III WRKY transcription factors comprise a single WRKY domain and a C_2_H_2_ or C_2_HC zinc finger motif, respectively. Besides, subgroup II WRKY transcription factors can be further divided into five subgroups (IIa, IIb, IIc, IId, and IIe) [2,4,5]. The first characterized *WRKY* gene, *SPF1*, was found in sweet potatoes and belonged to the typical subgroup I WRKY transcription factor [6]. More and more *WRKY* genes were reported in different species, including *Arabidopsis thaliana* [7], *Gossypium hirsutum L.* [8], rice [9], castor bean [10], cassava [11], cucumber [12], maize [13], wheat [14], tomato [15], millet [16] and so on.

WRKY transcription factors play a critical role in adaptation to abiotic stress. The TaWRKY2 of wheat could bind with the promoters of *STZ* and *RD29B* to enhance wheat’s drought resistance [17]. The sweet sorghum SbWRKY50 could directly bind to the upstream promoters of *SOS1* and *HKT1* and involved in plant responses to salt by regulating ion homeostasis [18]. The *BcWRKY46* gene was strongly induced by low temperature and abscisic acid (ABA), which activated related genes in ABA signaling pathway, thereby improving cold tolerance in *Brassica nuciformis* [19]. WRKY transcription factors also play an essential role in biotic stress. *OsWRKY45* plays a pivotal role in *Magnaporthe grisea* resistance and rice plants’ resistance to *Magnaporthe grisea* was stronger after Os*WRKY45* overexpression [20]. OsWRKY53 could activate rice defenses against *Nilaparvata lugens* by activating an H_2_O_2_ burst and suppressing ethylene biosynthesis [21]. CsWRKY50, a WRKY subgroup Ⅱc transcription factor, positively regulates disease resistance to *Pseudoperonospora cubensis* in *Cucumis sativus* [22]. WRKY transcription factors also play an essential role in the secondary metabolism of plants. Maize transcription factor gene Z*mWRKY79* could regulate plant defensins biosynthesis in response to different stress conditions [23]. TcWRKY1 plays an important role in taxol biosynthesis in *Taxus chinense* [24]. The overexpression of *A. thaliana* *AtWRKY18*, *AtWRKY40* and *AtMYC2* significantly increased the accumulation of abietane diterpenes which resistant to various bacteria and fungi in *Salvia sclarea* [25]. WRKY transcription factors also play an essential role in plant growth and development. In *A. thaliana*, At*WRKY46* plays a dual role in regulating plant response to drought and salt stress and opening light-dependent stomata in guard cells [26]. AtWRKY46 also regulated the development of lateral roots of *A. thaliana* under osmotic or salt stress by regulating the expression of abscisic acid and auxin related genes [27]. *Triticum aestivum* L. TaWRKY51 was a key factor that functions in lateral roots formation. 1-aminocyclopropane-1-carboxylic acid synthase (ACS) genes involved in ethylene biosynthesis. TaWRKY51 inhibited the expression of ACS genes by binding to the W-box cis-element present in their promoter region [28]. *A. thaliana* AtWRKY36 could promote the elongation of hypocotyls by directly inducing the expression of *ELONGATED HYPOCOTYL 5* (*HY5*) [29]. MdWRKY9 caused dwarf in apple plants by directly inhibiting the expression of the brassinolide (BR) rate-limiting synthetase *Dwarf4* (*MdDWF4*) [30].

Cotton fiber development is a complex process, which consists of five successive but overlapping stages: (i) fiber initiation: from 3 days before anthesis to 5 days after anthesis (−3 DPA-5 DPA); (ii) rapid elongation and primary wall synthesis (5–16 DPA); (iii) transition from primary wall synthesis to secondary wall synthesis (16–20 DPA); (iv) secondary wall synthesis (20–40 DPA); (v) maturation (40–50 DPA) [31]. The cotton fiber is a single-cell tubular protuberance differentiated from ovule trichome cells. The initial stage of cotton fiber is the critical period determining cotton yield because it determines how many ovule trichome cells develop into fibers. About 25% to 30% of the ovule trichome cells differentiate into cotton fibers [32]. The regulation mechanism of cotton fiber development is similar to that of *A. thaliana* trichomes development [33]. The gene *Gl2* encodes a HD-ZIP (homeodomain/leucine-zipper) IV domain transcription factor in *A. thaliana* [34]. TTG2 encodes a WRKY transcription factor similar to GL2 in the fuction of regulating trichome growth [35]. With the publication of cotton genome sequences [36,37,38,39], the researchers analyzed the whole genome of the *WRKY* gene family in *G. raimondii* and *G. arboreum* and found that there were 112 *WRKY* genes in *G. raimondii* and 109 *WRKY* genes in *G. arboreum*. The transcriptome analysis of these genes indicates that the *WRKY* gene might play an essential role in cotton fiber development [40]. The researchers identified 239 *WRKY* genes in *G. hirsutum* L. TM-1, of which 15 belonged to the subgroup Ⅱa *GhWRKY* genes. The expression patterns of type Ⅱa *GhWRKY* genes in different tissues and leaves during senescence were analyzed, and the function of *GhWRKY17* was verified. It was found that its overexpression in *A. thaliana* enhanced *A. thaliana* to resist senescence [41]. The researchers also identified the subgroup Ⅱd *GhWRKY* genes of *G. hirsutum L.* TM-1, identified 34 Ⅱd *GhWRKY* genes, analyzed the expression profile of subgroup Ⅱd *GhWRKY* genes under drought and salt stress, screened ten high expressed genes, and verified the function of *Gh_A11G1801*. Through virus-induced gene silencing (VIGS) of *Gh_A11G1801* in *G. hirsutum* L., it was found that *G. hirsutum* L. plants’ resistance to drought stress was reduced [42]. Recent studies have shown that the group Ⅱd gene *GhWRKY16* played a role in fiber initiation and elongation by regulating the expression of downstream target genes *GhHOX3*, *GhMYB109*, *GhCESA6D_D11* and *GhMYB25* which were related to cotton fiber development [43].

At present, there were few reports on the function of the *WRKY* gene in cotton fiber development, so we have taken the WRKY transcription factor as the research target to study its possible role in cotton fiber development. We first used molecular biology and bioinformatics methods to analyze putative group III *WRKY* genes in *G. hirsutum* L. Then we selected the *GhWRKY53* which was highly expressed in the fiber initiation stage for cloning and functional identification. Isolation and identification of critical genes regulating cotton fiber initiation are of great significance to understand the molecular mechanism of cotton fiber formation and guide the molecular improvement of cotton fiber.

## 2. Results

### 2.1. Identification and Chromosome Distribution of Putative Group III WRKY Genes in G. hirsutum L.

**Figure 1:**
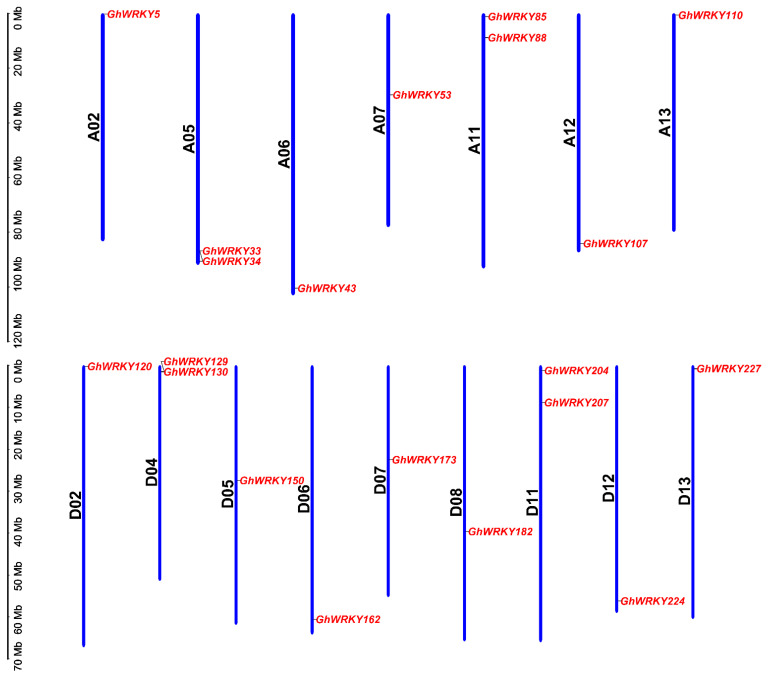
Chromosomal distribution of putative group III *WRKY* genes in *G. hirsutum* L. The scale represented megabases (Mb). The chromosome IDs are indicated beside each vertical bar. The *GhWRKYs* are displayed on different chromosomes. Blue bars represented the length of the chromossomes in *G. hirsutum* L.

According to the previous study [41], it had been found that there was 21 putative group III *WRKY* genes in *G. hirsutum* L. (Supplementary Table S1). We indentified the chromosome positions of putative group III *WRKY* genes in *G. hirsutum* L. by by CottonFGD (https://cottonfgd.org/, accessed on 1 April 2021) and displayed the chromosome distribution of putative group III *WRKY* genes in *G. hirsutum* L. by TBtools (Figure 1). The map showed that 21 candidate genes were located on 16 chromosomes, 9 genes on subgenome A and 11 genes on subgenome D. *GhWRKY69* (*Gh_A08G2417*) was located on scaffold2012_A08. The localized chromosomes were A02/05/06/07/11/12/13 and D02/04/05/06/07/08/11/12/13. The chromosomes A01/03/04/08/09/10 and D01/03/09/10 did not contain putative group III *WRKY* genes. The number of genes on the mapping chromosomes ranged from 1 to 2, of which 12 chromosomes had only one gene and 4 chromosomes contain 2 genes. We searched the characteristics of putative group III *WRKY* genes in *G. hirsutum* L., including genomic DNA length (bp), coding sequence CDS length (bp), coding sequence GC content (%), coding protein length (aa), exon number, gene start and termination position on the chromosome, isoelectric point (PI), protein molecular weight (MW) by CottonFGD (https://cottonfgd.org/, accessed on 1 April 2021). They are shown in Supplementary Table S1. The genomic DNA length of these candidate genes ranged from 1084 bp (*GhWRKY34*) to 2498 bp (*GhWRKY130*). The CDS length of the coding sequence ranged from 828 bp (*GhWRKY43*) to 1122 bp (*GhWRKY130*). The GC contents of the coding sequences ranged from 38.9% (*GhWRKY5*) to 45.4% (*GhWRKY129*). All genes contained three exons; the isoelectric point was between 4.894 and 7.964. The proteins’ molecular weight ranged from 31.185 kDa (*GhWRKY162*) to 40.785 kDa (*GhWRKY107*). The prediction of subcellular localization by the Wolf-PSORT (https://wolfpsort.hgc.jp/, accessed on 1 April 2021) showed that they were located on the nucleus (Supplementary Table S1). *GhWRKY53* was located in the region 29771353 to 29772620 of the A07 chromosome. The genome DNA length of *GhWRKY53* was 1268 bp. There were three exons. The GC content was 42.5%. The isoelectric point (pI) was 5.164. The predicted molecular weight was 38.182 kDa (Supplementary Table S1).

### 2.2. Phylogenetic Analysis of Putative Amino Acid Sequences from the III WRKY Group

**Figure 2:**
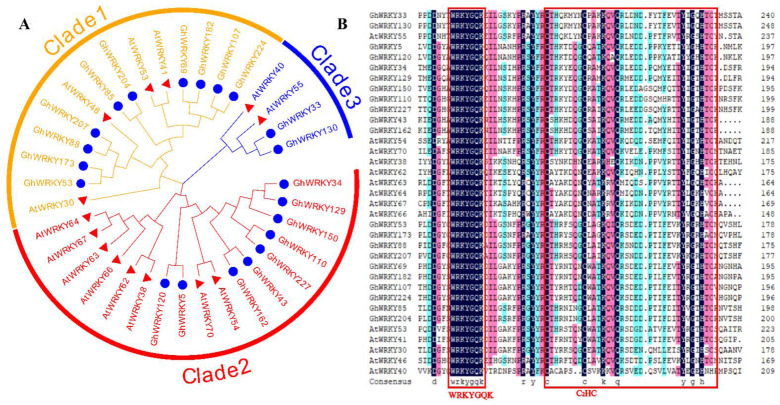
Phylogenetic analysis. (**A**) The phylogenetic tree of the amino acid sequences of putative group III WRKY amino acid sequences in *G. hirsutum* L. and *A. thaliana*, which was constructed by MEGA X software with the neighbor-joining (NJ) method with 1000 bootstrap replicates. GhWRKY amino acid sequences are marked by the blue circles; AtWRKY amino acid sequences are marked by the red triangles. (**B**) Multiple alignment of the amino acid sequences of putative group III WRKY in *G. hirsutum* L. and *A. thaliana*. The red box represents the conserved domain (WRKYGQK) and the C_2_HC zinc finger motif of putative group III WRKY amino acid sequences in *G. hirsutum* L. and *A. thaliana*, respectively.

To further analyze the evolutionary relationship of putative amino acid sequences from the III WRKY group, we constructed a phylogenetic tree using the amino acid sequences of 21 putative group III WRKY proteins in *G. hirsutum* L. (Figure 2). The results of multiple alignment indicated that the amino acid sequences of putative group III WRKY proteins in *G. hirsutum* L. were highly similar, with the typical WRKY domain and C_2_HC zinc finger motif. The phylogenetic tree is divided into three clades, the first clade had 10 putative amino acid sequences, the second clade had 9 putative amino acid sequences, and the third clade had 2 amino acid sequences. Among them, the putative amino acid sequences in subgenome A and its homologous putative amino acid sequences in subgenome D were well clustered in one clade, such as GhWRKY53 and GhWRKY173, GhWRKY88 and GhWRKY207, GhWRKY69 and GhWRKY182, GhWRKY107 and GhWRKY224, GhWRKY85 and GhWRKY204, GhWRKY5 and GhWRKY120, GhWRKY43, and GhWRKY162, GhWRKY110 and GhWRKY227, GhWRKY33 and GhWRKY130. Besides, the homologous putative amino acid sequences of GhWRKY150, GhWRKY34, and GhWRKY129 were clustered together, of which GhWRKY34 and GhWRKY129 had the highest homology. At the same time, GhWRKY150 was the homologous putative amino acid sequence of GhWRKY34 in subgenome D. The putative amino acid sequence of GhWRKY53 belonged to subgenome A of *G. hirsutum* L. The putative amino acid sequence of GhWRKY173 is homologous to GhWRKY53 in subgenome D (Figure S1). Then, we downloaded the amino acid sequences of *A. thaliana* putative group III WRKY proteins through the UniProt database and carried out multiple alignment and phylogenetic tree construction with putative amino acid sequences from the III WRKY group in *G. hirsutum* L. The Figure 2 demonstrated that putative group III WRKY amino acid sequences have the conserved domain (WRKYGQK) and the C_2_HC zinc finger motif, which are specific in subgroup III WRKY amino acid sequences (Figure 2).

### 2.3. Structure and Conserved Domain Analysis of Putative Group III WRKY Genes in G. hirsutum L.

**Figure 3:**
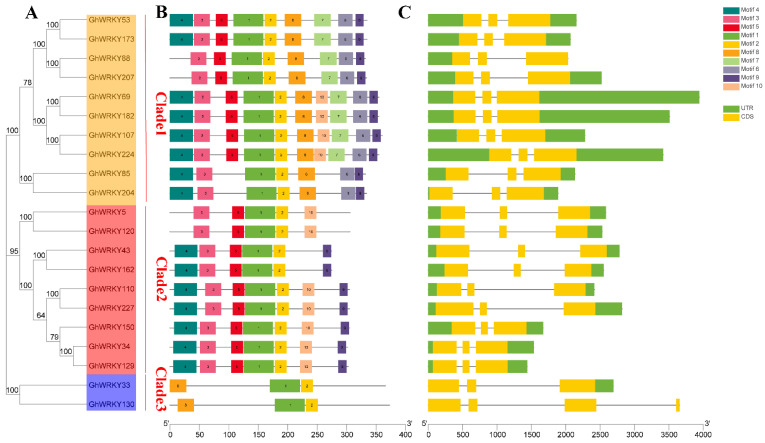
Phylogenetic relationship, motifs, and gene structures of group III *WRKY* genes or proteins in *G. hirsutum* L. (**A**) Phylogenetic analysis of putative amino acid sequences from the III WRKY group in *G. hirsutum* L. which was constructed by using MEGA X software with the neighbor-joining (NJ) method with 1000 bootstrap replicates; (**B**) Conserved motifs of group III WRKY proteins in *G. hirsutum* L. analyzed by the MEME; (**C**) The gene structures of group III *WRKY* genes in *G. hirsutum* L. which was performed by Gene Structure Display Server (GSDS). The CDSs, untranslated regions (UTRs) and introns are indicated with yellow rectangles, blue rectangles, and black lines, respectively.

We analyzed the gene structure and conserved domain of putative group III WRKY genes in *G. hirsutum* L. We plotted the phylogenetic tree, gene structure, and conserved domain map together with the phylogenetic tree (Figure 3). As shown in the figure, most genes had similar exon-intron structure and gene length. They all had three exons. Exclude *GhWRKY130* which had three introns, the other genes had two introns. Exclude *GhWRKY130* had no UTR and *GhWRKY33* had only the 3′-UTR, the other genes had UTR regions. Through the analysis of conserved domains, it was found that all genes had Motif1 and Motif2. Exclude *GhWRKY33* and *GhWRKY130*, all genes had Motif3. Exclude *GhWRKY88*, *GhWRKY207*, *GhWRKY5*, *GhWRKY120*, *GhWRKY33* and *GhWRKY130*, all genes had Motif4. Exclude *GhWRKY85*, *GhWRKY204*, *GhWRKY33* and *GhWRKY130*, all genes had Motif5. The motif information and the multiple alignment information showed that Motif1 and Motif2 were the highly conserved WRKY domain (WRKYGQK) and the C_2_HC zinc finger motif in putative group III *WRKY* genes in *G. hirsutum* L.

### 2.4. Analysis of Tissues Expression Pattern of Putative Group III WRKY genes in G. hirsutum L.

**Figure 4:**
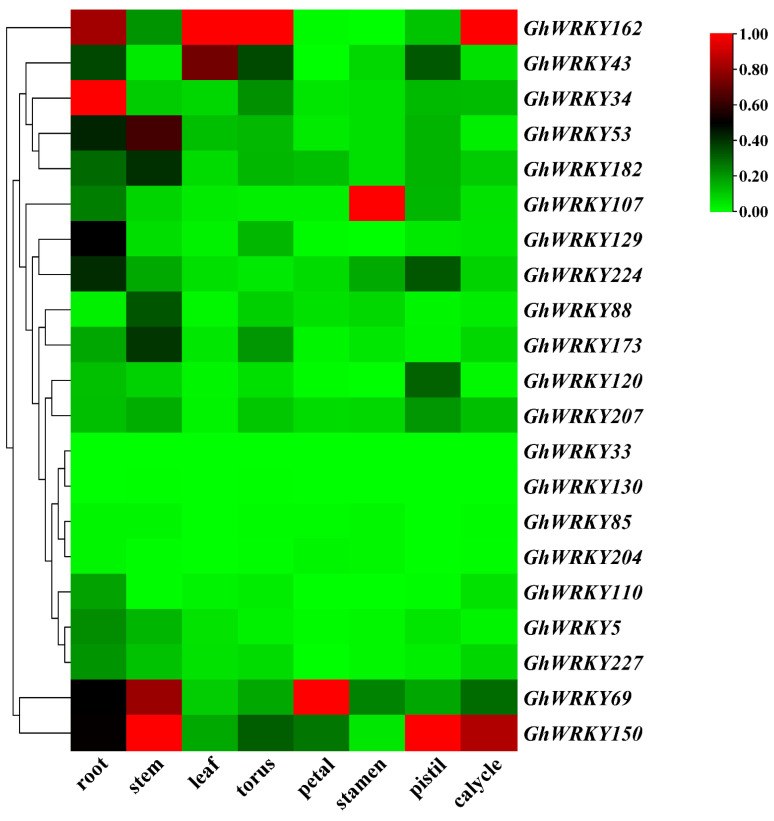
Expression profiles of group III *WRKY* genes in various tissues of *G. hirsutum* L. TM-1.

*G. hirsutum* L. tissue expression pattern analysis showed no prominent expression of *GhWRKY33* and *GhWRKY130* genes in different tissues, while the other 20 genes showed different expression patterns in various tissues (Figure 4). The expression level of 12 of the 21 genes was relatively low, and the fragments per kilobase million (FPKM) of all tissues was less than 50. The other 9 genes were highly expressed in specific tissues, and the FPKM was more than 50 or even more than 428. *GhWRKY34* (FPKM = 93.3), *GhWRKY53* (FPKM = 58.6), *GhWRKY69* (FPKM = 72.7), *GhWRKY129* (FPKM = 59.4), *GhWRKY150* (FPKM = 135.1), *GhWRKY162* (FPKM = 428.7), *GhWRKY173* (FPKM=87.4), *GhWRKY182* (FPKM = 59.1) were highly expressed in the petal. Among them, *GhWRKY34* (FPKM = 51.9) was highly expressed in roots. *GhWRKY43* (FPKM = 55.4) and *GhWRKY162* (FPKM = 76.3) were highly expressed in leaves; *GhWRKY150* (FPKM = 100) and *GhWRKY162* (FPKM = 118.2) were highly expressed in calyx; *GhWRKY69* (FPKM = 59.5) and *GhWRKY150* (FPKM = 74.3) were highly expressed in the stem. The gene *GhWRKY53* we studied was markedly expressed in root (FPKM = 22.3), stem (FPKM = 47.2), and torus (FPKM = 58.6) (Supplementary Table S2).

### 2.5. Analysis of the Expression Pattern of Putative Group III WRKY Genes in G. hirsutum L. during Fiber Development

**Figure 5:**
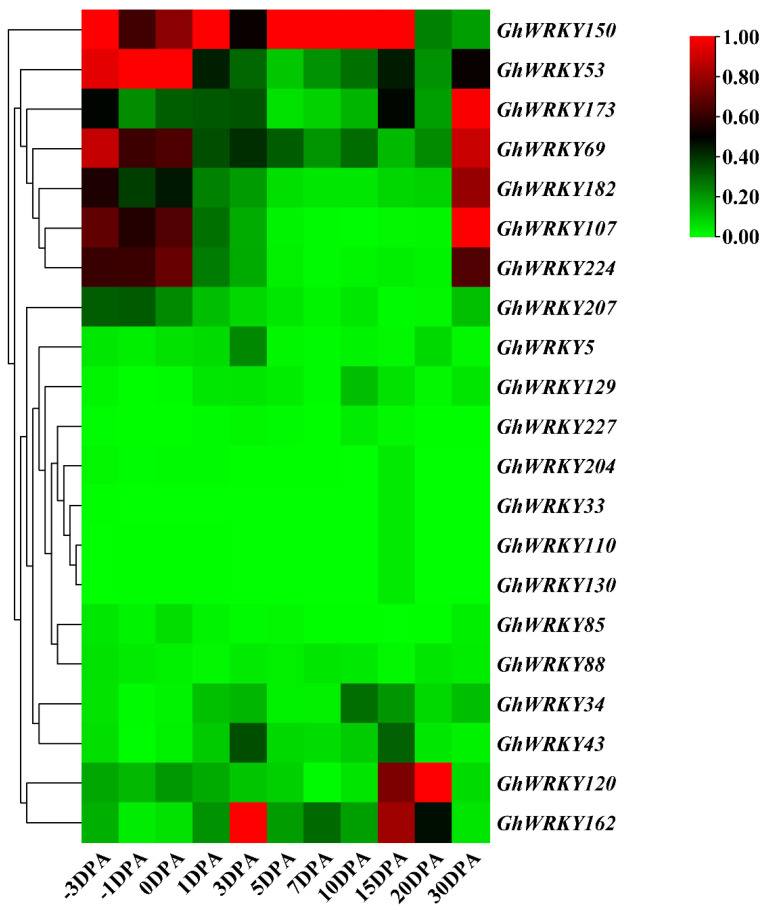
Expression profiles of group III *WRKY* genes during fiber development in *G. hirsutum* L. TM-1.

**Figure 6:**
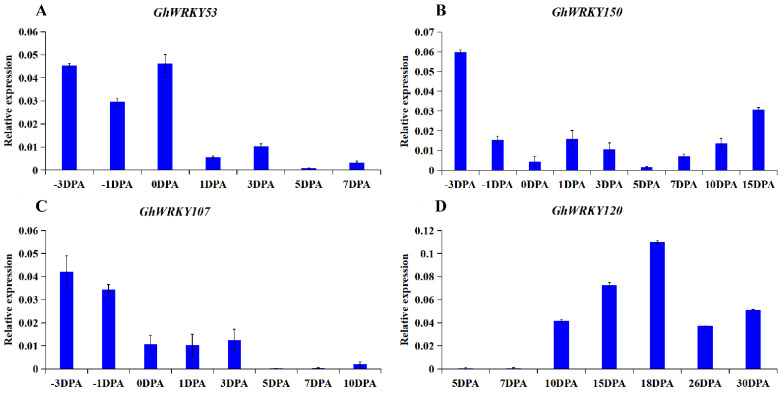
The qRT-PCR results of (**A**) *GhWRKY53* (**B**) *GhWRKY150* (**C**) *GhWRKY107* and (**D**) *GhWRKY120* during the fiber development in *G. hirsutum* L. TM-1. Note: −3 DPA to 30 DPA indicated by −3, −1, 0, 1, 3, 5, 7, 10, 15, 18, 26, 30 DPA. The variance bars showed the standard deviation of three biological replicates.

To explore the role of putative group III WRKY genes in fiber development, fiber development transcriptome analysis was carried out on ovules or fibers of *G. hirsutum* L. at different stages. The heatmap was drawn (Figure 5). Taking the FPKM value greater than one as the screening condition for gene expression in various stages of ovule or fiber development, we found that 5 members of the 21 members were basically not expressed in ovule and fiber tissues, and 76% of the members were expressed in ovule and fiber tissues. Besides, 13 of the 21 genes’ expression levels were relatively low, and the FPKM of all stages was less than 20. Seven genes, *GhWRKY53*, *GhWRKY69, GhWRKY107*, *GhWRKY150*, *GhWRKY173*, *GhWRKY182*, and *GhWRKY224*, had higher expression levels at the fiber initiation stage (−3 DPA–1 DPA) and fiber maturation stage (30 DPA). *GhWRKY34* was markedly expressed in 1 DPA and 30 DPA, FPKM > 10. *GhWRKY120* was expressed markedly in 0 DPA and 1 DPA, and FPKM > 10. *GhWRKY207* was expressed markedly in −3 DPA −1 DPA and 30 DPA, FPKM > 10 (Supplementary Table S3). To sum up, most of putative group III WRKY genes were expressed markedly at fiber initiation and fiber maturation, which play an essential role in fiber initiation and maturation. We further verified the expression pattern of *GhWRKY53*, *GhWRKY150*, *GhWRKY107* and *GhWRKY120* during cotton fiber development, using the ovules or fiber cDNA of −3 DPA, −1 DPA, 0 DPA, 1 DPA, 3 DPA, 5 DPA, 7 DPA, 10 DPA, 16 DPA, 18 DPA and 30 DPA as templates for real-time fluorescence quantitative PCR (qRT-PCR). The results were shown in Figure 6. It can be seen from the figure that the expression trend of *GhWRKY53* was consistent with the transcriptome data. *GhWRKY53* was expressed markedly at the initiation of cotton fiber, and it might be involved in the biological process of cotton fiber initiation.

### 2.6. Clone of GhWRKY53 and Sequence Analysis

**Figure 7:**
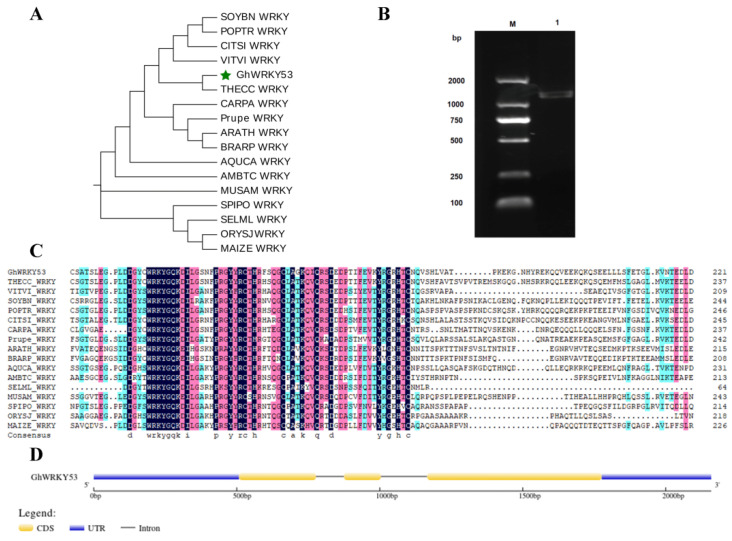
Sequence analysis of *GhWRKY53*. (**A**) The phylogenetic was constructed with amino acid sequences of *GhWRKY53* and orthologous proteins of GhWRKY53 in several other species (GhWRKKY53 is marked as a green pentagram); (**B**) Clone of *GhWRKY53* by RT-PCR. Note: Lane M: Marker 2000 bp, Lane 1: *GhWRKY53*; (**C**) Multiple alignment of *GhWRKY53* with orthologous proteins of other species; THECC: *theobroma cacao*, VITVI: *vitis vinifera*, SOYBN: *glycine max*, POPTR: *populus trichocarpa*, CITSI: *citrus sinensis*, CARPA: *carica papaya*, PRUPE: *prunus persica*, ARATH: *A. thaliana*, BRARP: *brassica rapa*, AQUCA: *aquilegia coerulea*, AMBTC: *amborella trichopoda*, SELML: *selaginella moellendorffii*, MUSAM: *musa acuminata*, SPIPO: *Spirodela polyrhiza*, ORYSJ: *oryza sativa*, MAIZE: *zea mays*. (**D**) The schematic diagram of the gene structure of *GhWRKY53*.

To further study the function of the *GhWRKY53* (*Gh_A07G1274*) gene, we cloned and verified its function. The accession number of *GhWRKY53* in NCBI was KJ825876. *GhWRKY53* was located in the region 29771353 to 29772620 of the A07 chromosome. The genomic DNA was 1268 bp in length and had three exons. The CDS length was 1008 bp. The GC content of CDS was 42.5%, the electric point (pI) was 5.164, and the predicted molecular weight was 38.182 kDa. The orthologous genes of *GhWRKY53* in other species were obtained from CottonFGD (https://cottonfgd.org/, accessed on 1 April 2021) database, and their amino acid sequences were downloaded. The multiple alignment and phylogenetic tree results showed that these genes had the conserved domain (WRKYGQK) and the C_2_HC zinc finger motif, specific in subgroup III *WRKY* genes. The *GhWRKY53* was closest to the evolution of the WRKY gene in tobacco. The structure of *GhWRKY53* contained 3 exons, 2 introns, and 2 UTRs (Figure 7).

### 2.7. Subcellular Localization of GhWRKY53

**Figure 8:**
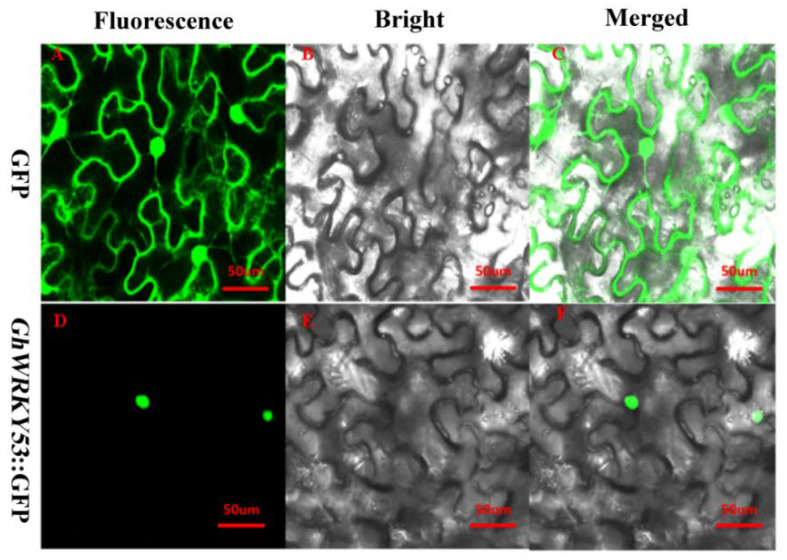
Subcellular localization of *GhWRKY53*. (**A**–**C**) Subcellular localization in tobacco leave cell, which was agroinfiltrated with pRI101-GFP vector. Fluorescence was observed in the whole cell, showing nucleus and membrane localization of GFP. (**D**–**F**) Subcellular localization in tobacco leave’s cell, which was agroinfiltrated with *GhWRKY53*-pRI101-GFP vector. Fluorescence was observed only in the nucleus, showing nuclear localization of *GhWRKY53*-GFP. Scale bar, 50 μm.

Transcription factors usually play a role in the nucleus. The subcellular localization result showed that fluorescence was observed only in the *Nicotiana tabacum* nucleus when the expression vector was *GhWRKY53*-pRI101-GFP. This specific localization, compared to pRI101-GFP alone, which was expressed through the whole cell. So *GhWRKY53* was located in the nucleus. This also corresponded to the nature of *GhWRKY53*, acting as a transcription factor in the nucleus (Figure 8).

### 2.8. Identification of Mutant AtWRKY46 in A. thaliana

**Figure 9:**
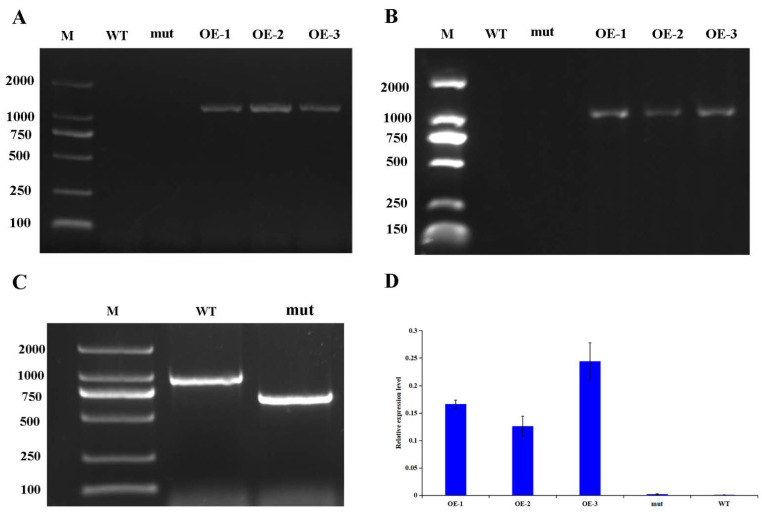
PCR analysis of the transgenic plants of *GhWRKY53*. (**A**) The results of DNA identification of *GhWRKY53*. in transgenic *A. thaliana*; (**B**) The results of cDNA identification of *GhWRKY53* in transgenic *A. thaliana*; (**C**) Identification of *AtWRKY46* mutant in *A. thaliana*. Primers: LP, RP and BP. (**D**) qPCR identification of *GhWRKY53* in transgenic *A. thaliana*. Note: Lane M: Marker 2000 bp; WT: Amplification of GhWRKY53 gene in wild type *A. thaliana*; mut: Amplification of *AtWRKY46* gene in corresponding mutant of *AtWRKY46* gene in *A. thaliana*; OE: Amplification of GhWRKY53 gene in transgenic lines of *GhWRKY53* in *A. thaliana*. The variance bars showed the standard deviation of three biological replicates.

*The corresponding homologous gene of GhWRKY53 in *A. thaliana* is AtWRKY46 (AT2G46400).* When the genomic DNA of wild type *A. thaliana* was used as template and LP and RP were used as primers for PCR amplification, the target band was 1129 bp. Because the length of T-DNA reaches several thousand KB, in the genomic DNA of *A. thaliana* mutant, if the mutant is a pure mutant, the full-length sequence can not be successfully obtained when LP and RP are used as primers. Only primers BP and RP can amplify, and the sequence length will be significantly shorter than the target sequence amplified with LP and RP as primers in wild type. If the mutant is heterozygous, when the mutant DNA is used as template for PCR amplification, in addition to the sequence amplified by BP and RP as primers, LP and RP primers will also amplify the same sequence as in wild-type A. thaliana. We used LP + RP + BP as primer for PCR amplification in A. wild-type and mutant genomic DNA, and then detected the amplification products by agarose gel electrophoresis. It was found that the band amplified in *A. thaliana* wild-type (WT) DNA was about 1000 bp, and that in *A. thaliana* mutant (mut) DNA was about 750 bp. In Figure 9C the band of lane WT on the gel corresponds to the amplification of wild type *A. thaliana* AtWRKY46 *gene*. The band of lane mut on the gel is special present in the *A. thaliana* mutant and corresponds to the amplification of corresponding mutant of AtWRKY46 gene in A. thaliana. Therefore, *A. thaliana* mutant was a pure mutant, which can be used in follow-up experiments (Figure 9D).

### 2.9. Genetic Transformation and Phenotypic Analysis of A. thaliana

**Figure 10:**
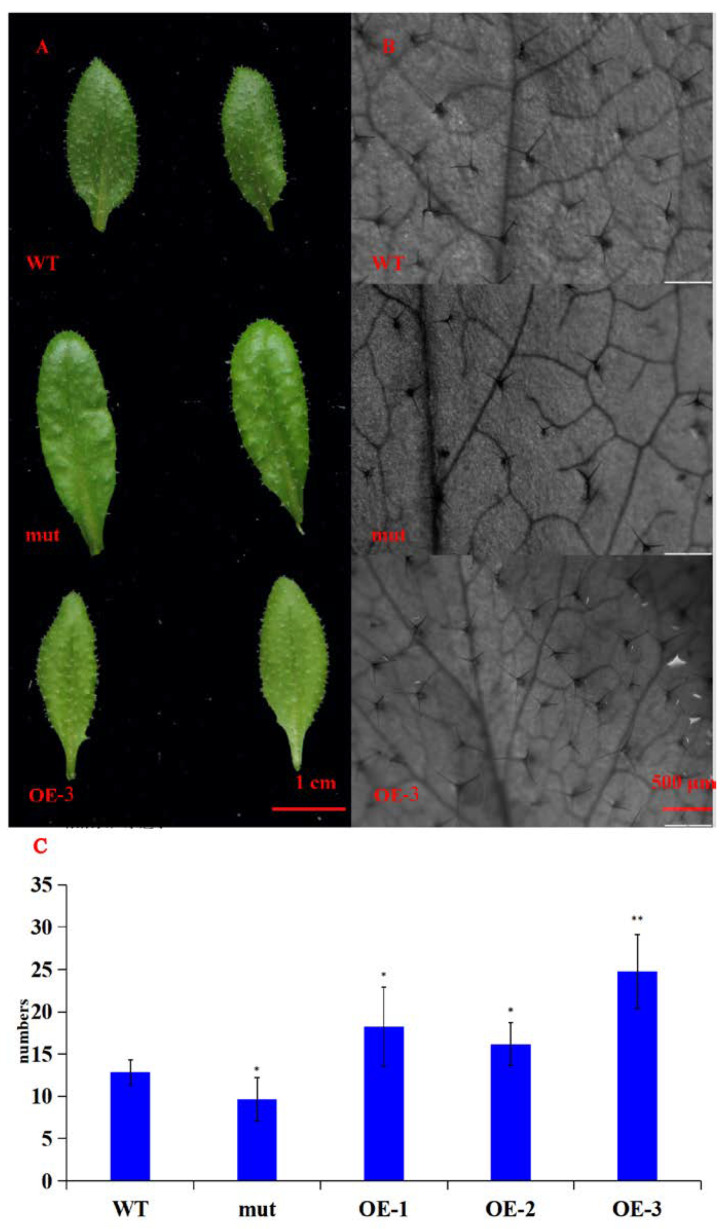
Phenotypic identification of transgenic *A. thaliana*. (**A**) The rosette leaves of wild type *A. thaliana* (WT), mutant *A. thaliana* (mut), and transgenic T5 generation *A. thaliana* line 3 (OE-3) under camera (bar, 1 cm); (**B**) The pictures of transgenic T5 *A.* OE-3 (OE), wild type *A. thaliana* (WT) and mutant *A. thaliana* (mut) leaves under the stereomicroscope (bar, 500 μm, and area, 38 mm^2^); (**C**) The statistical data of the number of trichomes of transgenic T5 *A. thaliana* (OE-1, OE-2, OE-3), wild type *A. thaliana* (WT) and mutant *A. thaliana* (mut). The results showed that the number of trichomes of *GhWRKY53* transgenic lines was significantly more than that of the wild type and the mutant in *A. thaliana*. The significance of difference was analyzed with two tailed *t* test (* 0.01 < *p* < 0.05, ** *p* < 0.01). Data are represented as average values with SD.

Through the screening of transgenic *A. thaliana* positive seedlings, three T5 transgenic positive plants were obtained, numbered *GhWRKY53*-1 (OE-1), *GhWRKY53*-2 (OE-2), and *GhWRKY53*-3 (OE-3). The rosette leaves of the wild type of *A. thaliana*, the mutants of *GhWRKY53* in *A. thaliana* (N667992), and three *GhWRKY53* T5 transgenic *A. thaliana* lines OE1, OE2, and OE3 were selected for DNA and RNA extraction and cDNA synthesis. According to the CDS sequence of *GhWRKY53*, the DNA of transgenic *A. thaliana* was identified by primer PRI101 (35s)-F/*GhWRKY53*-pRI101-R (EcoRI) (Supplementary Table S4). The amplification results were shown in Figure 9A. The target band length was consistent with that of the reference sequence *GhWRKY53* in the database. The cDNA was detected by internal reference primer Actin-2-F/Actin-2-R. We designed PRI101 (35s)-F/*GhWRKY53*-pRI101-R (EcoRI) as a full-length cDNA amplification primer and *GhWRKY53*-qPCR-F/*GhWRKY53*-qPCR-R for qRT-PCR identifications. The identification results are shown in Figure 9B, which demonstrated that the electrophoretic bands of the full-length cDNA were consistent with the target bands. The expression of *GhWRKY53* in *A. thaliana* transgenic lines was prominent higher than that in wild type and mutants (Figure 9D). The above results revealed that *GhWRKY53* had been transformed into the wild-type *A. thaliana* and expressed normally in *A. thaliana*. The rosette leaves of three lines of the T5 transgenic *A. thaliana*, wild-type *A. thaliana*, and mutant were selected, and their trichomes’ number were compared and analyzed by stereomicroscope. The results hinted that the number of trichomes trichomes of *GhWRKY53* transgenic lines was significantly greater than that of the wild type and the mutant per unit area (38 mm^2^) in *A. thaliana*. The results are shown in Figure 10. It was speculated that *GhWRKY53* might play a key role in *A. thaliana* trichome occurrence and growth. Previous literature reported that *A. thaliana* and *G. hirsutum* L. had similarities in the process of trichomes development [33], so *GhWRKY53* might play an essential role in cotton fiber initiation, which was consistent with the specific expression of *GhWRKY53* at the fiber initiation stage of *G. hirsutum* L. TM-1.

### 2.10. Screening of GhWRKY53-Interaction Proteins by Yeast Two-Hybrid

A total of 42 positive clones were obtained through library screening. After removing repeat sequences, 12 potential interacting proteins were identified (Supplementary Additional File 2). And the identified protein sequences are shown in full in Supplementary Additional File 2. Functional annotation analysis showed that these proteins were mainly transcription factors of the MYB family, microtubule-associated proteins involved in microtubule binding, the B2 protein involved in programmed cell death, the heat shock protein DnaJ, the ubiquitin-related protein UBQ10, the calcium-binding related protein VSR3, and the abscisic acid (ABA)-related protein AGP30. The results suggested that *GhWRKY53* might be an essential node in fiber development cross-regulation by various biochemical pathways.

### 2.11. GhWRKY53 Interacted with GhRAX3

**Figure 11:**
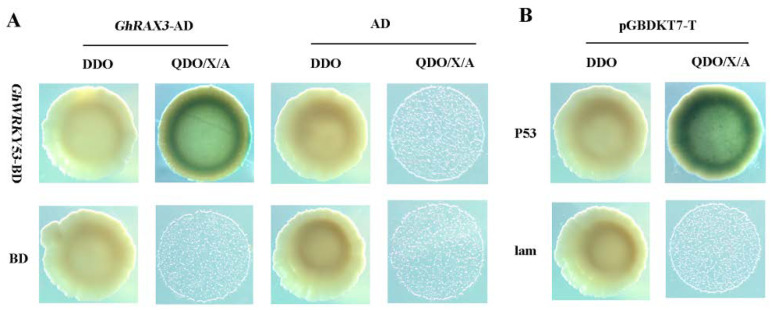
Yeast two-hybrid assay of GhWRKY53-GhRAX3 interaction. (**A**) Physical interaction of GhRAX3 with GhWRKY53 in yeast cells and the yeast co-transformed *GhWRKY53*-pGBKT7 + GhRAX3-pGADT7 grown on SD-Trp-Leu-His-Ade/X-α-Gal/AbA (QDO/X/A) medium. (**B**) pGBKT7-P53 + pGADT7-T and pGBKT7-lam + pGADT7-T were used as positive and negative controls, respectively. The yeast co-transformed the positive control vector grown on SD-Trp-Leu-His-Ade/X-α-Gal/AbA (QDO/X/A) medium. *GhWRKY53*-BD: *GhWRKY53*-pGBKT7, *GhRAX3*-AD: GhRAX3-pGADT7, BD: pGBKT7, AD: pGADT7, BD: pGBKT7, P53: pGBKT7-P53, T:pGADT7-T,lam:pGBKT7-lam, DDO: SD-Trp-Leu, QDO/X/A: SD-Trp-Leu-His-Ade/X-α-Gal/AbA.

In *A. thaliana*, R2R3 MYB type gene GL1 regulated trichome differentiation in *A. thaliana* [44]. TTG1, which encoded WD40 protein, and bHLH type transcription factor GL3/EGL3 could form MYB-bHLH-WD40 (MBW) ternary protein complex and activate downstream homeodomain/leucine zipper-type transcription factors *GL2* and *WRKY* class transcription factor *TTG2* to promote trichome growth [35]. GhRAX3 belongs to the MYB family of transcription factors, which might interact with *GhWRKY53* and participate in the growth and development of trichome. To confirm GhRAX3 protein interact with GhWRKY53 protein, GhRAX3 protein was selected and investigated using a Y2H system. The CDS of the GhRAX3 protein was cloned into the pGADT7 vector. Fusion construct plasmids pGADT7-GhRAX3 and pGBKT7-*GhWRKY27* were co-transformed into the yeast strain Y2HGold. Our results showed that the experimental group (*GhWRKY53*-pGBKT7+pGADT7-*GhRAX3*) and the positive control (pGBKT7-p53+pGADT7-T) grew well on SD-Trp-Leu (DDO) and SD-Trp-Leu-His-Ade/X-α-Gal/AbA (QDO/X/A) medium, and the yeast was blue. However, pGADT7+*GhWRKY53*-pGBKT7, GhRAX3-pGADT7+pGBKT7, pGADT7+pGBKT7, and the negative control pGBKT7-lam + pGADT7-T grew well on SD-Trp-Leu (DDO) medium but could not grow on SD-Trp-Leu-His-Ade/X-α-Gal/AbA(QDO/X/A) medium (Figure 11). The above results showed that the GhWRKY53 and GhRAX3 could interact with each other.

## 3. Discussion

Cotton is an important cash crop, which provides a large amount of natural fiber. As one of the most prominent transcription factor families in plants, members of the *WRKY* gene family play an essential role in many plant life processes, such as plant response to biotic and abiotic stresses, secondary metabolism of plants, and plant growth and development. With the continuous development of genome sequencing technology and functional genomics, it was possible to identify and analyze the whole plant genome family members. Up to now, WRKY transcription factors had been reported in detail in 79 plant species [45].

The release of the cotton genome sequence and the establishment of the cotton functional genome database CottonFGD provide a suitable research basis for identifying and analyzing *G. hirsutum L.* WRKY members. Several researchers had identified and analyzed cotton *WRKY* family genes in recent years and cloned and identified some essential genes [42,46,47,48,49,50]. However, there were few reports on the role of the *WRKY* gene in cotton fiber development. Heterologous expression of *GhWRKY15* in tobacco can affect plant growth and development, especially stem elongation [51]. *A. thaliana TTG2* encoded WRKY transcription factors, which play an important role in the differentiation of trichomes cell [52]. *Triticum aestivum* L. transcription factor TaWRKY51 was essitional in lateral root formation [28]. *A. thaliana AtWRKY36* can promote the elongation of hypocotyl [29]. *GhWRKY16* played a role in fiber initiation and elongation [43]. According to the previous identification of cotton *WRKY* genes [42], there are 21 putative group III *WRKY* genes in *G. hirsutum* L. We analyzed the expression patterns of putative group III *WRKY* genes in *G. hirsutum* L. during fiber development, and found that some of them were markedly expressed at the fiber initiation and maturation stages. The initial development process of cotton fiber determined the yield of cotton fiber. The mining of cotton fiber initiation genes would help us understand cotton fiber’s initial development process to improve cotton yield.

We selected the *GhWRKY53* markedly expressed at the fiber initiation stage for cloning and functional verification. *GhWRKY53* is a orthologous gene of *A. thaliana AtWRKY46*. They are all group III WRKY genes. *AtWRKY46* was expressed in the whole primordium of lateral roots in the early stage of lateral root development, and then the expression was limited to the stele of mature lateral roots. In osmotic/salt stress conditions, lateral roots were significantly reduced after *AtWRKY46* mutation, while the overexpression of *AtWRKY46* enhanced lateral root development [27]. WRKY transcription factors WRKY46, WRKY54, and WRKY70 in *A. thaliana* subgroup III play an active role in plant growth regulated by brassinolide (BR) and inhibit the expression of drought response-related genes [45]. The above results suggested that *AtWRKY46* gene was involved in the development of lateral roots of *A. thaliana*. Cotton fiber is developed from ovule epidermal cells [32]. Therefore, the function of *GhWRKY53* gene may be similar to that of AtWRKY46 during cotton fiber development, which may increase the number of processes of trichome cells and then increase the yield of cotton fiber.To determine the biological function of *GhWRKY53*, we found that the trichomes of transgenic *A. thaliana* were significantly more than those of wild type and *AtWRKY46* mutants after heterologous expression of *GhWRKY53* in *A. thaliana*. Studies had shown that the development of *A. thaliana* trichome was similar to that of cotton fiber [33]. Therefore, *GhWRKY53* may play an essential role in cotton fiber initiation.

To further explore the molecular mechanisms of *GhWRKY53*, we carried out a yeast two-hybrid experiment and finally screened 12 proteins that might interact with *GhWRKY53*. The screening library results showed that GhRAX3 belonged to the type of MYB transcription factor. In recent years, many studies have shown that transcription factor MYB plays an essential role in regulating the differentiation and development of *A. thaliana* trichome and cotton fiber [33,53]. It had been confirmed that MYB-related transcription factors could regulate the expression of WRKY-related genes in *A. thaliana* [35] and WRKY transcription factors could regulate the expression of MYB-related genes in *G. hirsutum* L. [43]. Therefore, GhRAX3 was most likely to interact with GhWRKY53 and participate in cotton fiber initiation. Whether transcription factors WRKY and MYB can interact with each other to promote fiber development has not been reported. We confirm the interaction of GhRAX3 protein with GhWRKY53 protein by using the Y2H system confirm. *GhRAX3* belongs to the R2R3 family of transcription factors, including SANT/MYB domain and homeobox domain. Its homologous gene in *A. thaliana* is *AtMYB36* (AT5G57620.1) [54]. In *A. thaliana*, *AtMYB36* is related to the differentiation of root endodermis and the synthesis of lignin [55,56]. In sea island cotton, *GbRL2*, which is homologous to *G. hirsutum* L. *GhRAX3*, is expressed at the initiation and early elongation stages (0 DPA, 3 DPA, 5 DPA and 8 DPA) and may be involved in the development of various organs of sea island cotton [57]. Therefore, we will focus on the interaction between GhRAX3 and GhWRKY53 in the future. The specific mechanism of the interaction between GhWRKY53 and GhRAX3 and the critical function of *GhWRKY53* in the initial stage of cotton fiber need to be further studied in *G. hirsutum* L. lines with *GhWRKY53* overexpression and knockout. Our research work will provide new ideas and methods for studying the biological function of putative group III *WRKY* genes and the process of cotton fiber development in *G. hirsutum* L.

## 4. Materials and Methods

### 4.1. Plant Materials and Strains

The cotton plant material used in this experiment was *G. hirsutum* L. standard line TM-1, which was planted in May 2019 in the experimental field of Cotton Research Institute of Chinese Academy of Agricultural Sciences, Anyang City, Henan Province, China. We used *Agrobacterium tumefaciens* LBA4404 as the transformation strain, Colombian wild type *A. thaliana* was used as the plant transformation material for subcellular localization experiment, and *N. tabacum* (benthamiana) was used as the plant transformation material for genetic transformation experiment. The corresponding mutant N667392 of the *GhWRKY53* in *A. thaliana* was purchased from the Arabidopsis Information Resource. (http://www.arabidopsis.org/, accessed on 1 April 2021). The seeds were sterilized with 1% sodium hypochlorite and 75% alcohol and sowed on MS solid medium and placed in a laboratory with light and a constant temperature provided by an available eletrothermal incubator. (*A. thaliana* seeds should be placed in a refrigerator at 4 °C for 48 h for vernalization). Two weeks later, they were transplanted into a nutrition bowl.The photoperiod was established with a temperature of 21 °C during the 16 h light period and of 18 °C during the 8 h dark period. *A. tumefaciens* transformed strain LBA4404, *Escherichia coli* transformed strain DH5 α, and chemically competent cell yeast transformed strain (Y2HGold, and Y187) were purchased from Weidi Biotechnology Co., Ltd. (Shanghai, China).

### 4.2. Identification and Sequence Analysis of Putative Group III WRKY Genes in G. hirsutum L.

Gu et al. identified 21 putative group III *WRKY* genes in *G. hirsutum* L. [41] by phylogenetic analysis using the *G. hirsutum* L. sequences (http://www.cottongen.org, accessed on 1 April 2021) [39] in the *G. hirsutum* L. functional gene database. In this study, 21 putative group III *WRKY* genes in *G. hirsutum* L. were used as subjects. Using the information provided by *G. hirsutum* L. functional genome database CottonFGD (https://cottonfgd.org/, accessed on 1 April 2021) [54], the biochemical parameters of putative group III *WRKY* genes in *G. hirsutum* L. were determined including genomic DNA length (bp), coding sequence CDS length (bp), coding sequence GC con-tent (%), coding protein length (aa), exon number, gene start and termination position on the chromosome, isoelectric point (PI) and protein molecular weight (MW). In this study, information related to genome, transcript, protein, and genome functional annotation of *G. hirsutum* L. TM-1 was downloaded on CottonFGD. They were determined by Nanjing Agricultural University in 2015 [39].

### 4.3. Chromosomal Location and Subcellular Location Prediction of Putative Group III WRKY Genes in G. hirsutum L.

The chromosome location information of putative group III *WRKY* genes in *G. hirsutum* L. was obtained from *G. hirsutum* L. functional genome database CottonFGD (https://cottonfgd.org/, accessed on 1 April 2021) [54], and the chromosome location information was visualized by TBtools software. We used the Wolf-PSORT (https://wolfpsort.hgc.jp/, accessed on 1 April 2021) [58] to predict putative group III *WRKY* genes in cotton (*G. hirsutum* L.).

### 4.4. Sequence Alignment and Phylogenetic Analysis of Putative Group III WRKY Genes in G. hirsutum L.

The amino acid sequences of putative group III WRKY proteins in *A. thaliana* were downloaded from UniProt to analyze the phylogenetic relationship. Multiple sequence alignment of the identified amino acid sequences of putative group III WRKY proteins in *G. hirsutum* L. and *A. thaliana* was carried out by Clustal W software (Uji, Kyoto, JPN) [59] and DNAMAN software (Lynnoti Biosofi, Quebec, Canada). And phylogenetic analyses based on the identified amino acid sequences of putative group III WRKY proteins in *G. hirsutum* L. and *A. thaliana* were constructed with the MEGA X software (The Pennsylvania State University University Park, PA, USA) [60] using the neighbor-joining method. The number of bootstrap replicates was 1000, and the rest of the parameters were set as the defaults.

### 4.5. Analysis of the Structure and Motifs of Putative Group III WRKY Genes in G. hirsutum L.

The gene structure display server GSDS2.0 (http://gsds.gao-lab.org/, accessed on 1 April 2021) [61] was used to visualize the exons, introns, and UTRs positions of these genes. The conservative motifs of putative group III *WRKY* genes in *G. hirsutum* L. were analyzed by the MEME program [62]. The phylogenetic tree, gene structure, and conserved protein sequence map were drawn by TBTools software [63]. The files used were the MAST file on the MEME website (https://meme-suite.org/meme/, accessed on 1 April 2021), the NWK file for phylogenetic tree analysis, and the GFF3 file for *G. hirsutum* L. genome annotation information.

### 4.6. Transcriptome Data-based Gene Expression Analyses in G. hirsutum L. Tissues

Raw RNA-Seq data for *G. hirsutum* L. root, stem, leaf, torus, petal, stamen, pistil, and calycle were downloaded from the NCBI Sequence Read Archive (https://www.ncbi.nlm.nih.gov/bioproject/PRJNA248163, accessed on 1 April 2021) [39]. The data were normalized using the reads per kilobase of exon model per million mapped reads(FPKM) algorithm. The expression heatmaps were visualized by Tbtools [63].

### 4.7. Gene Cloning and Sequence Analysis

The full-length cDNA of GhWRK53 was amplified from 0 DPA ovule cDNA of *G. hirsutum* L. TM-1 using *GhWRKY53*-pRI101-F (SalI)/*GhWRKY53*-pRI101-R (EcoRI) as primers. The amplified product was inserted into the overexpression vector pRI101, and the recombinant plasmid was transferred into the DH5α strain for sequencing. Using the GSDS2.0 (http://gsds.cbi, accessed on 1 April 2021) [61] showed the structure of *GhWRKY53*. All of primers used in this study were listed in Supplementary Table S4.

### 4.8. Sample Collection, DNA and RNA Extraction, cDNA Synthesis, Transcriptome Analysis, and qRT-PCR Analysis

Genomic DNA was extracted via the cetyl-trimethylammonium bromide (CTAB) method [64]. We collected the ovules of −3 DPA,−1 DPA, 0 DPA, 1 DPA, 3 DPA, and 5 DPA and the fibers of 7 DPA, 10 DPA, 15 DPA, 20 DPA, and 30 DPA of *G. hirsutum* L. TM-1. All samples were immediately frozen in liquid nitrogen and stored at-80 ℃. Plant total RNA was extracted by the RNAprep Pure Plant Plus Kit (Polysaccharides&Polyphenolics-rich) (DP441) (Tiangen, Beijing, China). The reverse transcription kit was the PrimeScript™ II 1st Strand cDNA Synthesis Kit (TAKARA, Dalian, China). It was used to reverse the extracted RNA to obtain the first-strand cDNA for transcriptomic and qRT-PCR analysis. The data were normalized using the reads per kilobase of exon model per million mapped reads (FPKM) algorithm. The expression heatmap was visualized by Tbtools [63]. The fluorescent quantitative kit was the MonAmp SYBR Green qPCR Mix (Monad, Suzhou, China). The data was calculated according to the 2^−ΔΔCT^ method [65]. The *G. hirsutum* L. His3 (GhHis3) and *A. thaliana* actin (AtActin2) genes were downloaded from CottonFGD (https://cottonfgd.org/, accessed on 1 April 2021) and Tair (https://www.arabidopsis.org/, accessed on 1 April 2021) respectively, which were used as reference controls. The related coding sequence information is shown in Supplementary Additional File 1. According to the candidate gene sequence, a relatively specific primer for real-time fluorescence quantitative PCR was designed by Primer3 software (Stanford, CA, USA) (Supplementary Table S4), and the amplification product of *GhWRKY53, GhWRKY150, GhWRKY107* and *GhWRKY120* were 201 bp, 156 bp, 98 bp and 190 bp respectively.

### 4.9. Subcellular Localization

The CDS sequence of *GhWRKY53* was amplified by using *GhWRKY53*-pBI101-GFP-F (SalI)/*GhWRKY53*-pBI101-GFP-R (BamHI) as primer and cDNA of ovule on the anthesis day (0 DPA) of *G. hirsutum* L. TM-1 as the template. The CDS sequence was inserted into the site of SalI and BamHI of the pRI101-GFP expression vector to construct the subcellular localization vector. By using *A. tumefaciens* transformation method, the constructed *GhWRKY53*-pRI101-GFP subcellular localization vector and the pRI101-GFP expression vector as the control were introduced into LBA4404 *A. tumefaciens*. Agrobacterium cells harboring the fusion protein genes were agroinfiltrated into leaves of *N. tabacum* through the abaxial surface, and 72 h later the samples were observed under aconfocal microscope (TCS SP8 STED 3X, Leica, Wetzlar, Germany).

### 4.10. Identification of A. thaliana Mutants

*A. thaliana* mutant is a T-DNA insertion mutant. Firstly, the T-DNA insertion information of *A. thaliana* (*AT2G46400*) was queried on the mutant information query website (http://signal.salk.edu/cgi-bin/tdnaexpress/, accessed on 1 April 2021). It was found that SALK_134310 (N667392) was a mutant passing through the exon region of *A. thaliana AT2G46400* gene. We purchased the mutant seeds at ABRC (https://abrc.osu.edu/, accessed on 1 April 2021) according to the accession number (SALK_134310.37.95.x) of the T-DNA insertion mutant provided on the website. Then, in the primer design website for identifying mutants (http://signal.salk.edu/tdnaprimers.2.html, accessed on 1 April 2021), we input the entry number (SALK_134310.37.95.x) of T-DNA insertion mutants to get two primers of LP (Left genomic primer) and RP (Right genomic primer), and query the BP universal primers on the home page of the website. Among them, LP is located on the upstream genome sequence of T-DNA, that is, the 5 ‘end. RP is located on the downstream genomic sequence of T-DNA, that is, the 3 ‘end. Another primer, BP, is located at the 5 ‘end of T-DNA. Finally, using the DNA of wild type *A. thaliana* and mutant *A. thaliana* as templates, three primers were directly added to a PCR reaction for PCR amplification. The relevant primer information is shown in Supplementary Table S4.

### 4.11. Transformation of A. thaliana and Phenotype Observation

The CDS sequence of *GhWRKY53* was amplified by using *GhWRKY53*-pRI101-F (SalI)/*GhWRKY53*-pRI101-R (EcoRI) as primer and cDNA of ovule on the anthesis day (0 DPA) of *G. hirsutum* L. TM-1 as the template. The CDS sequence was inserted into the site of SalI and EcoRI of the pRI101 expression vector to construct the overexpression vector. The overexpression vector *GhWRKY53*-pRI101 was transformed into *A. tumefaciens* LBA4404 and transformed into *A. thaliana* by *A. tumefaciens*-mediated flower soaking method, and the seeds of T0 generation were harvested. The transgenic *A. thaliana* T0 generation seeds were planted on the solid MS medium with Kanamycin (Kana) antibiotic concentration of 50 mg/mL, and the transgenic positive *A. thaliana* was screened. The early anthesis phenotypes of three transgenic lines, wild type, and mutants were analyzed. The trichomes of mature rosette leaves were observed. Seven rosette leaves were selected for each plant. After decolorization with ethanol, four regions of each leaf were randomly selected and observed with 3.2 times objective lens (area: 38 mm^2^) under the stereomicroscope, and the number of surface trichomes was counted.

### 4.12. Yeast Double Hybrid and Point-to-Point Verification

Total RNAs from *G. hirsutum* L. 0 DPA ovule were extracted using the RNAprep pure plant kit (TIANGEN, Shanghai, China) according to the manufacturer’s protocol. The resulting RNAs were treated with DNase I prior to synthesize cDNA with oligo (dT) primers and PrimeScript II RTase (Takara, Beijing, China). DPA DPA The cotton fiber development yeast two-hybrid (Y2H) cDNA library was constructed by using the cDNA of *G. hirsutum* L. 0 DPA ovule. The CDS sequence of *GhWRKY53* was amplified by using *GhWRKY53*-pGBKT7-F (EcoRI)/*GhWRKY53*-pGBKT7-R (BamHI) as primer and cDNA of ovule on the anthesis day (0 DPA) of *G. hirsutum* L. TM-1 as the template. The CDS sequence was inserted into the site of EcoRI and BamHI of the pGBKT7 expression vector to construct the bait vector. According to the manufacturer’s protocol (Clotech), we performed library screening. The *GhWRKY53*-pGBKT7 bait vector and cDNA library were co-transformed into yeast Y2HGold competent cells. The transformed yeast cells were initially screened on SD-Trp-Leu (DDO)/X/A medium and cultured in the 30 ℃ electrothermal constant temperature incubator (Opple lighting, Zhongshan, GZ, China) for 3–5 days, and the positive clones were screened more strictly on QOD/X medium. The blue clone was extracted by Yeast Plasmid Mini Preparation Kit (Beyotime, Shanghai, China). We sequenced the vector from the blue clone and compared it with *G. hirsutum* L.’s reference genomics to identify the protein interacting with *GhWRKY53*. The CDS sequence of *GhRAX3* was amplified by using GhRAX3-pGADT7-F (EcoRI)/GhRAX3-pGADT7-R (BamHI) as primer and cDNA of ovule on the anthesis day (0 DPA) of *G. hirsutum* L. TM-1 as the template. The CDS sequence was inserted into the site of EcoRI and BamHI of the pGADT7 expression vector to construct the prey vector. The positive control vector plasmids were pGBKT7-p53 + pGADT7-T, and the negative control vector plasmids were pGBKT7-lam + pGADT7-T. Other control vectors plasmids were pGADT7 + *GhWRKY53*-pGBKT7, GhRAX3-pGADT7 + pGBKT7 and pGADT7 + pGBKT7. The fusion expression vector plasmids were GhRAX3-pGADT7 + *GhWRKY53*-pGBKT7. They were co-transformed into yeast Y2HGold, respectively. The transformed yeast cells were grown on SD-Trp-Leu (DDO) and SD-Trp-Leu (DDO)/X/A medium and cultured in the 30 ℃ electrothermal constant temperature incubator (Opple lighting, Zhongshan, GZ, China) for 3–5 days.

## 5. Conclusions

To sum up, our study analyzed the members of putative group III *WRKY* genes in *G. hirsutum* L. Further, we verified the function of the *GhWRKY53* in *A. thaliana* by the heterologous expression method. We found that the *GhWRKY53* plays an essential role in increasing the density of *A. thaliana* trichomes, and a MYB transcription factor-like protein GhRAX3, to which it interacts, was identified.

## Data Availability

The data presented in this study are available in the article and Supplementary Material.

## Supplementary Materials

The following are available online at https://www.mdpi.com/article/10.3390/plants10061235/s1, Additional File 1: The coding sequences of the reference control genes, Additional File 2: Amino acid sequences of 12 potential interacting proteins with GhWRKY53 protein, Figure S1: Phylogenetic analysis of putative amino acid sequences from the III *WRKY* group in *A. thaliana*, Table S1: Identification of putative group III *WRKY* genes in *G. hirsutum* L., Table S2: The FPKM values of putative group III *WRKY* genes in *G. hirsutum* L. tissues, Table S3: The FPKM values of putative group III *WRKY* genes in *G. hirsutum* L. during fiber development, Table S4: A list of primers used in this study.

## Abbreviations

AAT	amino acid transporter
−3 DPA	Ovule at minus 3 day post anthesis
−1 DPA	Ovule at minus 1 day post anthesis
0 DPA	Ovule at 0 day post anthesis
1 DPA	Ovule at 1 day post anthesis
3 DPA	Ovule at 3 day post anthesis
5 DPA	Ovule at 5 day post anthesis
7 DPA	Fiber at 7 day post anthesis
10 DPA	Fiber at 10 day post anthesis
15 DPA	Fiber at 15 day post anthesis
18 DPA	Fiber at 18 day post anthesis
26 DPA	Fiber at 26 day post anthesis
20 DPA	Fiber at 20 day post anthesis
30 DPA	Fiber at 30 day post anthesis.
*G. hirsutum* L.	*Gossypium hirsutum* L.
*A. thaliana*	*Arabidopsis thaliana*
*A. tumefaciens*	*Agrobacterium tumefaciens*
*N. tabacum*	*Nicotiana tabacum*
RT qPCR	Reverse transcription quantitative real-time PCR
FPKM	Fragments per kilobase million
