# Supplementary material for: Identification of the Group III WRKY Subfamily and the Functional Analysis of GhWRKY53 in Gossypium hirsutum L
Source: Plants (Basel). 2021 Jun 17;10(6):1235. doi: 10.3390/plants10061235 (PMC8233714; doi:10.3390/plants10061235)
Supplement: Supplementary file 1 [file Figure S1.pdf › Supplementary files/Figure S1.pdf]

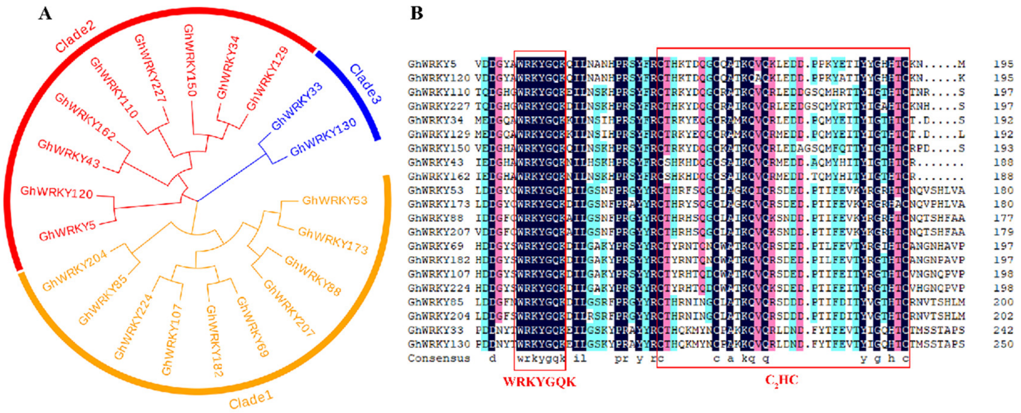

Figure S1 Phylogenetic analysis of putative amino acid sequences from the III *WRKY* group in *A. thaliana.* (A) The phylogenetic tree of putative group Ⅲ WRKY genes in *G. hirsutum* L. which was constructed by MEGA Ⅹ software with the neighbor-joining (NJ) method with 1000 bootstrap replicates, the GhWRKY53 protein marked by the green pentagram; (B) The result of multiple alignment of putative group Ⅲ WRKY protein sequences in *G. hirsutum* L. The red box represents the conserved domain (WRKYGQK) and the C_2HC zinc finger motif of putative group III WRKY amino acid sequences in *G. hirsutum* L., respectively.
