# Supplementary material for: Identification of the Group III WRKY Subfamily and the Functional Analysis of GhWRKY53 in Gossypium hirsutum L
Source: Plants (Basel). 2021 Jun 17;10(6):1235. doi: 10.3390/plants10061235 (PMC8233714; doi:10.3390/plants10061235)
Supplement: Supplementary file 1 [file plants-10-01235-s001.zip › Supplementary files/Additional file1.pdf]

>Actin-2 (AT3G18780.2)
ATGGCTGAGGCTGATGATATTCAACCAATCGTGTGTGACAATGGTACCGGTATGGTGAA
GGCTGGATTTGCAGGAGATGATGCTCCCAGGGCTGTTTTTCCCAGTGTTGTTGGTAGGC
CAAGACATCATGGTGTCATGGTTGGGATGAACCAGAAGGATGCATATGTTGGTGATGA
AGCACAATCCAAGAGAGGTATTCTTACCTTGAAGTATCCTATTGAGCATGGTGTTGTTA
GCAACTGGGATGATATGGAAAAGATCTGGCATCACACTTTCTACAATGAGCTTCGTATT
GCTCCTGAAGAGCACCCTGTTCTTCTTACCGAGGCTCCTCTTAACCCAAAGGCCAACA
GAGAGAAGATGACTCAAATCATGTTTGAGACCTTTAACTCTCCCGCTATGTATGTCGCC
ATCCAAGCTGTTCTCTCCTTGTACGCCAGTGGTCGTACAACCGGTATTGTGCTGGATTC
TGGTGATGGTGTGTCTCACACTGTGCCAATCTACGAGGGTTTCTCTCTTCCTCATGCCA
TCCTCCGTCTTGACCTTGCTGGACGTGACCTTACTGATTACCTCATGAAGATCCTTACA
GAGAGAGGTTACATGTTCACCACAACAGCAGAGCGGGAAATTGTAAGAGACATCAAG
GAGAAGCTCTCCTTTGTTGCTGTTGACTACGAGCAGGAGATGGAAACCTCAAAGACCA
GCTCTTCCATCGAGAAGAACTATGAATTACCCGATGGGCAAGTCATCACGATTGGTGCT
GAGAGATTCAGATGCCCAGAAGTCTTGTTCCAGCCCTCGTTTGTGGGAATGGAAGCTG
CTGGAATCCACGAGACAACCTATAACTCAATCATGAAGTGTGATGTGGATATCAGGAAG
GATCTGTACGGTAACATTGTGCTCAGTGGTGGAACCACTATGTTCTCAGGTATCGCTGA
CCGTATGAGCAAAGAAATCACAGCACTTGCACCAAGCAGCATGAAGATTAAGGTCGTT
GCACCACCTGAAAGGAAGTACAGTGTCTGGATCGGTGGTTCCATTCTTGCTTCCCTCA
GCACATTCCAGCAGATGTGGATCTCCAAGGCCGAGTATGATGAGGCAGGTCCAGGAAT
CGTTCACAGAAAATGTTTCTAA

>Gh_D03G0370.1 DESC:Histone H3.3 ; ORG:Gossypium hirsutum; CHR:D03;
ATGGCCCGTACCAAGCAAACCGCCCGTAAGTCTACTGGTGGGAAGGCTCCAAGGAAG
CAACTTGCTACCAAGGCTGCCCGTAAATCTGCCCCAACCACCGGTGGTGTGAAGAAGC
CTCATCGATACCGTCCTGGAACTGTTGCTCTTCGTGAAATTCGTAAATACCAGAAGAGT
ACTGAGCTTCTTATCAGGAAATTGCCTTTCCAGAGGCTTGTTCGTGAAATTGCCCAGGA
CTTCAAGACTGATTTGCGTTTCCAGAGCCATGCTGTTCTAGCTCTCCAGGAAGCTGCAG
AGGCATACCTTGTGGGTCTTTTTGAAGACACCAACCTTTGCGCGATCCATGCCAAGCGT
GTCACAATTATGCCCAAGGACATCCAGTTGGCTCGTAGGATCAGGGGAGAGCGTGCTT
AA
