# Supplementary material for: Identification of the Group III WRKY Subfamily and the Functional Analysis of GhWRKY53 in Gossypium hirsutum L
Source: Plants (Basel). 2021 Jun 17;10(6):1235. doi: 10.3390/plants10061235 (PMC8233714; doi:10.3390/plants10061235)
Supplement: Supplementary file 1 [file plants-10-01235-s001.zip › Supplementary files/Additional file2.pdf]

>Gh_A03G1606.1 NAME:VSR3; DESC:Vacuolar-sorting receptor 3 ; ORG:Gossypium hirsutum; CHR:A03;
MEIRRLHILLLLQGFMLFSVIGNCYARFVVEKNSLRVTSPEKIKGTYDSAIGNFGIPQYG
GSMAGAVVYPKENQKGCKSFDDFGISFKSKPGALPTFVLVDRGDCFFALKVWNVQQAGA
SAVLVADDIQEALITMDTPEEDRLAAKYIENITIPSALIEKNFGEILKKAISGGDMVNVNL
DWRESVPHPDDRVEYELWTNSNDECGVKCDMLMEFLKDFKGAAQILEKGGYTQFTPHYI
TWYCPQAFSLSRQCKSQCINYGRYCAPDPEQDFSSGYEGKDVVIENLRQLCVFKVANETN
KPWLWWDYVTDFQIRCPMKEKKYNKECADVVIRALGLDGKKIEKCMGDPNADEDNPVL
KEEQEAQVGKGSRGDVTILPTLVVNDRQYRGKLAKGAVLKAICAGFEETTEPAVCLSGDV
ETNECLDNNGGCWQDKATNLTACKDTFRGRVCECPLVDGVQFKGDGYSHCEASGSGRC
KINNGGCWHEARDGHAYSACLDDGNGKCQCPPGFKGDGVKNCEDIDECKEKKACQCPE
CSCKNTWGSYECTCSGDLLYIRDHDTCISKSGTEVRSSWAAVWVILIGLAMASGGAYLIY
KYRLRSYMDSEIRAIMAQYMPLDSQAEVPNHVSDGRA

>Gh_A05G1816.1 NAME:APT1; DESC:Adenine phosphoribosyltransferase 1, chloroplastic ; ORG:Gossypium hirsutum; CHR:A05;
MLQRALIFSSSNPICSQLPTTAPPDNTVFPKRNGGWIIPFSSYQLPAFRFRNYRRTLLPP
LCSGNVFDSAANRPNMASQDEQDPRISNISSAIRVIPDFPKPGIIFQDITTLLLDTKAFR
DTIDLFVERYRGKDISVVAGIEARGFIFGPPIALAIGAKFVPMRKPNKLPGEVLSEEYSL
EYGKDTMEMHVDAVKAGERALIIDDLVATGGTLSAAIRLLECVGVHVVECACVIELPELK
GRERLGGKPLFVLLSST

>Gh_A05G2799.1 NAME:TOR1; DESC:Microtubule-associated protein TORTIFOLIA1 ; ORG:Gossypium hirsutum; CHR:A05;
MSFKNRSPTTILQPQPQDLKQRVYTCLNKLADRDTLAVASAELDSIARNLTADSISPFLN
CLYGTDSSSKSPVRRQCVCLLTLLSRSHGNALSPHLSKMVSTLSRRLRDPESAVRSACVE
ATAAMSSHITKPPFSVLSKPLIEMLVVEQDVNSQVGAAMCLAAAIDAAPDPESEQLRKVL
PKLGKLVRNESFKAKAAVFGVIGSVASVGGAGSKGVLDWLVPCAVDALSSEDWATRKAA
AEALGKVAMAEKELTAEFKSACVVALENKRFDKVKIVRETFNRSLDLWKEVPGVCEVSAP
SQSDSSSVETGNGSFGCFPSVTKSVDDAGFRTLQSKKVVPTSRSPPSDASTEPTAEKETHL
SSNYRNWNISDFSKLDRTKPSDCKTEIAEPRKSLFFKAYDNIKNGDLGVMESREIGDSRD
SRLETKRVLFAKVRDENVQKYGGLKTGSRVVPFHDENLDFDDKKAAVEADENPKEVEDL
SLIREQLAQIEDQQSNLLNLLQKFIGSSQSGINSLETRVNGLEMALDEISYDLSLSNGRIP
NMDSADKTCCKLPGTEFLSPKFWRKPEGRFSTSRLSSSGRMLSLNAAHNIPDKDSRAETY
IPAVSQRHQHQGCGGFIFPVADGCNGITDNSGSSNRLSKNTIQNAERVQLGNGSDPDGTT
LVRCTAPMKP

>Gh_A06G0385.1 NAME:Dnajb13; DESC:DnaJ homolog subfamily B member 13 ; ORG:Gossypium hirsutum; CHR:A06;
MVNHLNQEPVPIFNQKKKGFSLREITRVLRSWRLRLKKSSSSDQPIYQPSQGSPDENGDG
QKIVRVSSWTSDSDGSRSEGRSSKGFFKYRSMDACFSRQDSTNSKGTSKRSRSPSPSPKP
SSQNKEGSLRRSTTTDRNGLFEPLSGSTSRPNGNPIMFSNSTGVVKPPPIEKQLECTLED
LCYGCTEKIKIKRDVITESGQRVEHKEMLSINVEPGWKKGTKITFEGMGNEVPRLYAADV
TFVIAEKQHPIFGRDGDDLELTIEIPLVKALTGCSLPIPLLGGEKMELKIDEIIHPGYEK

IITGQGMPTTKEAASRGNLKVRFLINFPTELTDDQQAAAVRILGDSSS

>Gh_A13G0991.1 NAME:NAGK; DESC:Acetylglutamate kinase, chloroplastic ; ORG:Gossypium hirsutum; CHR:A13;
MGATATGKSLHFCSSQDPLSFLSLTMTKSKAETLNLNFRFSKPPLTPNSRRLRLSIRASS
PSSTALNDPPLPSLTNSDLQLRVRILSESLPYIQKFRGKTIVVKYGGAAMKSAELKASVV
RDLVLLSCVGLRPVLVHGGGPEINVLLNQLNIPAQFRDGLRVTDARTMEVVSMVLVGKVN
KDLVSRINYAGATAVGLSGMDGRLLVARPSPNAAQLGFVGEVARVDPTVLQTIVDNGSIP
VIASVAADEYGQPYNINADTVAGEVAAALGAEKLILLTDVAGILRNREDPGSLVKEIDIK
GVKKMIEEGKVGGGMIPKVNCCIRSLDQGVTTASIIDGRVEHSLLHEIMTDEGAGTMITG

>Gh_D01G0585.1 NAME:UBQ10; DESC:Polyubiquitin 10 ; ORG:Gossypium hirsutum; CHR:D01;
MQIFVKTLTGKTITLEVESSDTIDNVKAKIQDKEGIPPDQQRLIFAGKQLEDGRTLADYN
IQKESTLHLVLRLRGGMQIFVKTLTGKTITLEVESSDTIDNVKAKIQDKEGIPPDQQRLI
FAGKQLEDGRTLADYNIQKESTLHLVLRLRGGMQIFVKTLTGKTITLEVESSDTIDNVKA
KIQDKEGIPPDQQRLIFAGKQLEDGRTLADYNIQKESTLHLVLRLRGGMQIFVKTLTGKT
ITLEVESSDTIDNVKAKIQDKEGIPPDQQRLIFAGKQLEDGRTLADYNIQKESTLHLVLR
LRGGMQIFVKTLTGKTITLEVESSDTIDNVKAKIQDKEGIPPDQQRLIFAGKQLEDGRTL
ADYNIQKESTLHLVLRLRGGMQIFVKTLTGKTITLEVESSDTIDNVKAKIQDKEGIPPDQ
QRLIFAGKQLEDGRTLADYNIQKESTLHLVLRLRGGFRMVF

>Gh_D03G1692.1 NAME:dnaJ; DESC:Chaperone protein DnaJ ; ORG:Gossypium hirsutum; CHR:scaffold3874_D03;
MAIIPCGSTFVAQWHIRPQLTTRSYVPNRIMTARLGVTSTMSYLRASNSGLFARDSLPLL
SFVRPSQTSHHRRGAQFIVRAETDYYTVLGVSRNASKSEIKSAYRKLARSYHPDVNKDPG
AETKFKEISNAYEVLSDDEKRSLYDKYGEAGLKGAGMGMGDFSNPFDLFESLFEGMGGM
GGMGMGGRSSRNRAVDGQDEYYSLVLNFKDAVFGVEKEIEITRLESCGTCNGSGAKPGTT
PSKCTTCGGQGQVISSARTPLGVFQQVMTCSSCGGTGEISTPCNTCSGDGRVRRTKRISLK
VPAGVDSGSRLRVRSEGNAGRRGGSAGDLFVVIEVIPDPVLKRDDTNILYTCKVSYIDAI
LGTTIKVPTVDGMVDLKIPAGTQPNTTLVMAKKGVPVLNKTNMRGDQLVRVQVEIPKRL
SSEEKKLIEELADLSKGKTASSRR

>Gh_D05G0050.1 NAME:RAX3; DESC:Transcription factor RAX3 ; ORG:Gossypium hirsutum; CHR:D05;
MGRAPCCDKANVKKGPWSPEEDAKLKAYIEQYGTGGNWIALPQKIGLKRCGKSCRLRW
LNYLRPNIKHGGFSEEEDNIICSLYISIGSRWSIIAAQLPGRTDNDIKNYWNTKLKKKLLGR
RKQPSNIHRLSNQDPNDPHQPTGSDDNQFSQGLSNSAMERLQLHMQLQTLQNPFSFYNNP
ALWPKIHPLQEKMIQNMQASNGNPNLLMQPLLPPNPQPANEQSTVHQQDYPEISNTKGLE
GLDNCLDGISPSDGSVPFGNGDNLMDSTSTAAAVQQVSSFQGELDVFLNNKSSTTGGYPQ
EDQMVNQLDCFRDINGSKDSLIWWPTDFDAKSASSNSWDSTSVLQSNGMFQDFELGYPM

>Gh_D05G1532.1 NAME:AGP30; DESC:Non-classical arabinogalactan protein 30 ; ORG:Gossypium hirsutum; CHR:D05;

MGFAVVVLVVKAVLLVQLSLLLLSTFTVSAPIWPQASPPHYHAVSPVVPPSHPPTHHHHH
HPHPHPHPHPPTKPPTPTPPPVHPPPKAPVQPPTKPPTSAPPKPPVQPPTKPPTKPPTQP
PPKPPTQPPTKPPTKPPTQSPTKPPTHPPSHPPAKPPTSSHVAVQGVVYCKSCKYAGVDT
LLGAKPIPRATVRLTCKDAKNELTVQFKTDKNGYFFLQAPITIYNFDLHNCSVSLVSSPL
RACSKPSNLNGGLKGAPLKPEKPSTSKKLPYVLYSVGPFAFEPTCHKN

>Gh_D05G2725.1 NAME:DNAJ1; DESC:DnaJ protein homolog ; ORG:Gossypium hirsutum; CHR:D05;
MFGRAPKKSDNTKYYEILGVPKTASQDDLKKAYRKAAIKNHPDKGGDPEKFKELAQAYE
VLSDPEKREIYDQYGEDALKEGMGGGGGAHDPFDIFQSFFGGNPFGGGGSSRGRRQRRG
EDVIHPLKVSLEDLYNGTSKKLSLSRNIICSKCKGKGSKSGASMKCSGCQGSGMKVSIRHL
GPSMIQQMQHPCNDCKGTGETINDKDRCPQCKGEKVVQEKKVLEVNVEKGMQNGQKIT
FPGEADEAPDTVTGDIVFVLQQKDHPKFKRKGDDLFVEHTLTLTEALCGFQFILTHLDGRQ
LLIKTHPGEVVKPDQFKAINDEGMPMYQRPFMRGKLYIHFTVDFPDSLAPEQCKALEAVL
PPRTSVQLTDMELDECEETTLYDVNIEEEMRRKQAQAAQEAYEEDDDMHGGAQRVQCA
Q

>Gh_D12G2323.1 DESC:B2 protein ; ORG:Gossypium hirsutum; CHR:D12;
MENNQQSFWQFSDQLRVQNSNLANLSLNDSIWSSGYVSKRPDERRNFDIRVGGELNSVNS
LKPKVTDFNSFSNDGWKIGATSNANIGFGPIGPIGSQNNVGVNGGINKGIYSKPGNNNNN
FNVNLKGSKNKGEDNHGTKSGKKNTNKKNNNNNDNNNENKDGKSAADKRFKTLPPSES
LPRNETVGGYIFVCNNDTMEENLRRQLFGLPPRYRDSVRAITPGLPLFLYNYSTHQLHGVF
EAASFGGTNIDPTAWEDKKCPGESRFPAQVRVITRKICEPLEEDSFRPILHHYDGPKFRLE
LNIPEALSLLDIFADQQDTCIS

>Gh_D13G1239.1 NAME:NAGK; DESC:Acetylglutamate kinase, chloroplastic ; ORG:Gossypium hirsutum; CHR:D13;
MGATATGKSLHFCSSQDPLSFLSLTKTKSKAETLNLNFRFSKPPLTPNSRRLRLSIRASS
PSSTALNDPPLPSLSNSDLQLRVRILSESLPYIQKFRGKTIVVKYGGAAMKSAELKASVV
RDLVLLSCVGLRPVLVHGGGPEINVLLNQLNIPAQFRDGLRVTDARTMEVVSMVLVGKVN
KDLVSRINYAGATAVGLSGMDGRLLVARPSPNAAQLGFVGEVARVDPTVLQTIVDNGSIP
VIASVAADEYGQPYNINADTVAGEVAAALGAEKLILLTDVAGILRNREDPGSLVKEIDIK
GVKKMIEEGKVGGGMIPKVNCCIRSLAQGVTTASIIDGRVEHSLLHEIMTDEGAGTMITG
